# Recent Changes in Land Water Storage and its Contribution to Sea Level Variations

**DOI:** 10.1007/s10712-016-9399-6

**Published:** 2016-11-15

**Authors:** Yoshihide Wada, John T. Reager, Benjamin F. Chao, Jida Wang, Min-Hui Lo, Chunqiao Song, Yuwen Li, Alex S. Gardner

**Affiliations:** 1 NASA Goddard Institute for Space Studies, 2880 Broadway, New York, NY 10025 USA; 2 Center for Climate Systems Research, Columbia University, 2880 Broadway, New York, NY 10025 USA; 3 Department of Physical Geography, Faculty of Geosciences, Utrecht University, Heidelberglaan 2, 3584 CS Utrecht, The Netherlands; 4 International Institute for Applied Systems Analysis, A-2361 Laxenburg, Austria; 5 NASA Jet Propulsion Laboratory, California Institute of Technology, Pasadena, CA 91109 USA; 6 Institute of Earth Sciences, Academia Sinica, Taipei, 11529 Taiwan; 7 Department of Geography, Kansas State University, 118 Seaton Hall, Manhattan, KS 66506 USA; 8 Department of Atmospheric Sciences, National Taiwan University, Taipei, 10673 Taiwan; 9 Department of Geography, University of California Los Angeles, 1255 Bunche Hall, Los Angeles, CA 90095 USA

**Keywords:** Land water storage, Sea level rise (SLR), Groundwater depletion (GWD), Reservoir impoundment, Climate variability

## Abstract

Sea level rise is generally attributed to increased ocean heat content and increased rates glacier and ice melt. However, human transformations of Earth’s surface have impacted water exchange between land, atmosphere, and ocean, ultimately affecting global sea level variations. Impoundment of water in reservoirs and artificial lakes has reduced the outflow of water to the sea, while river runoff has increased due to groundwater mining, wetland and endorheic lake storage losses, and deforestation. In addition, climate-driven changes in land water stores can have a large impact on global sea level variations over decadal timescales. Here, we review each component of negative and positive land water contribution separately in order to highlight and understand recent changes in land water contribution to sea level variations.

**Electronic supplementary material:**

The online version of this article (doi:10.1007/s10712-016-9399-6) contains supplementary material, which is available to authorized users.

## Introduction

Sea level rise (SLR) over the past century is generally attributed to increased ocean heat content (thermal expansion, e.g., Abraham et al. 2013) and increased rates of melt and solid ice discharge (calving) from glaciers and ice sheets (e.g., Gardner et al. 2013; Shepherd et al. 2012). Large-scale anthropogenic and natural changes in land water storage, defined as snow, surface water, soil moisture, and groundwater storage, excluding glaciers, have also contribute to observed rates of SLR on annual to centennial timescales (Milly et al. 2003; Syed et al. 2010; Reager et al. 2016).

Human transformations of Earth’s surface have impacted continental patterns of river flow and water exchange between land, atmosphere, and ocean, ultimately affecting global sea level variations. Massive impoundment of water in reservoirs and artificial lakes has reduced the outflow of water to the sea, while river runoff has increased due to excessive groundwater mining, wetland and endorheic lake storage losses, and deforestation (Chao et al. 2008; Wada et al. 2012a, b; Church et al. 2013). In the IPCC Fifth Assessment report (IPCC AR5), anthropogenic changes in terrestrial water storage (primarily filling of reservoirs and groundwater mining) were included in the sea level change budget but natural fluctuations were excluded due to poor knowledge of their change and the expectation that such changes would be small on decadal timescale. Recent work by Reager et al. (2016) showed that climate-driven changes in water stores (e.g., soil moisture and groundwater) can have a large impact on global sea level variations over decadal timescales.

Impoundment of fresh water behind reservoirs constructed in the 1950s through the 1980s resulted in increased storage of water on land resulting in a lowering of sea level. Over the past few decades, the rate of impoundment has been surpassed by increased human mining of groundwater reserves leading to a net increase in sea levels (Wada 2016). Better understanding of the temporal evolution of the contributing processes leading to change in total land water storage is critical to closing decadal sea level budgets and to understanding changes in rates of sea level change.

Around 8000 km^3^ of water is presently sequestered behind large reservoirs. More than 90% of this total reservoir capacity was created after the 1950s resulting in an cumulative decrease in the global sea level change of 30 mm (Chao et al. 2008). Other processes such as groundwater mining (or pumping of groundwater at rates exceeding the natural recharge rate), wetland and endorheic lake storage losses, and deforestation have led to an increase in the rates of land water contribution to the oceans. Water stored in wetlands and endorheic lakes (e.g., the Aral Sea and the Lake Urmia) has been heavily used for agricultural production leading to a net transfer of water from land to the ocean resulting in SLR. Deforestation reduces the infiltration capacity of the soil due to compaction by heavy logging, farm machinery, overgrazing and trampling by cattle and increases soil erosion. In newly cleared areas, runoff usually increases, especially in the rainy season, leading to greater chances of flooding. The combined effect of these processes has reduced soil moisture and groundwater reserves, increasing the rate of sea level change by 0.3–0.5 mm year^−1^ during a recent few decades (Church et al. 2013; Gregory et al. 2013; Wada et al. 2016).

Knowledge of how anthropogenic changes in land water storage affect future sea levels is poorly constrained. A simple extrapolation of recent trends is likely unrealistic for several reasons: (1) A major dam-building boom is underway across the developing world. An additional few hundred km^3^ water could therefore be sequestered on land, amounting to between 2 and 4% of present reservoir storage. This rate of dam building is likely to slow down in coming decades as suitable sites become scarcer and public opposition to environmental impacts of dams grows. (2) Deforestation may also decrease as forests are cleared from all but inaccessible sites and as stricter environmental regulations aim to preserve remaining forests. (3) Groundwater mining has been increasing due to excessive pumping in many irrigated regions (e.g., India, Pakistan, China, and Iran), and it is unclear whether such rates can be sustained into the future. All these unknowns lead to large uncertainties in projections of anthropogenic changes in land water storage.

Here we review each of the contributing process to changes in land water storage to provide understanding of recent trends in associated land water contribution to sea level change.

## Mechanisms of Land Water Contribution and Other Sea Level Components

Construction of reservoirs and resulting impoundment for power generation and water resource management results in increased land water storage through reservoir filling and raising of surrounding groundwater tables. A great quantity of water, which has been impounded behind dams (artificial reservoirs), would otherwise reside in the oceans rather than on land. Thus, each reservoir can be thought of as a “one-time” deduction of SLR. Impounded water is ultimately sourced from the ocean, resulting in a lowering of mean sea level (Chao et al. 2008). Groundwater pumping for agriculture (mostly irrigation) and other uses (industrial and municipal) contributes to SLR through the persistent removal of groundwater at rates exceeding natural recharge rates. The mined water finds its way to the ocean as increased surface runoff and/or evapotranspiration that later falls as precipitation over the ocean (Wada et al. 2016). Human water use is also a major driver for decreasing wetland and endorheic lake storage across the globe. In addition, forest clearing releases water stored in both biotic tissues and soil, which leads to positive SLR.

Anthropogenic influences, such as water use and land use change on the Earth’s land water storage, are clearly seen in the decreasing volume of the Aral sea (Pala 2006, 2011), decreased flows of the Colorado and Yellow Rivers (Gleick 2003), dwindling groundwater resources over intense irrigated regions such as the Ogallala aquifer (Scanlon et al. 2012a, b), the California’s Central Valley (Famiglietti et al. 2011), the North China Plain (Cao et al. 2013), northwest India and northeast Pakistan (Rodell et al. 2009; Tiwari et al. 2009), and the Tigris-Euphrates (Voss et al. 2013). The degree of aquifer depletion is reported at an alarming rate over the Indus, Saudi Arabia, Iran, northeastern China, the southwestern and Central USA, and northern Mexico (Rodell et al. 2009; Tiwari et al. 2009; Famiglietti et al. 2011; Konikow 2011; Döll et al. 2012, 2014; Scanlon et al. 2012a, b; Cao et al. 2013; Voss et al. 2013; Famiglietti 2014).

In addition to anthropogenic influences on land water storage, seasonal to decadal changes in climate can also modify the apparent SLR rate. Changes in the patterns of seasonal precipitation over the continents (e.g., the migration of the intertropical convergence zone during rainy seasons) and winter snow accumulation at high latitudes (e.g., Siberia and North America) can act to modulate global mean sea level and the annual amplitude of global mean sea level (Wouters et al. 2011). Multi-year variability in the distribution of water between the land and oceans due to El Niño–Southern Oscillation (ENSO) effects on weather and precipitation can result in droughts and/or raised groundwater tables over basin to continental scales that modify rates of ocean mass change (Boening et al. 2012; Syed et al. 2010; Reager et al. 2016).

Many complex processes contribute to generate net changes in global and regional sea levels, and the relative importance of each contributing term depends on the time period being analyzed. Many of the contributing processes are not stationary and can undergo substantial changes in magnitude or even direction at annual to decadal timescales.

## Groundwater Contribution

The rate of groundwater depletion (GWD) and its contribution to SLR has been subject to much debate (Gregory et al. 2013; Taylor et al. 2013). In the IPCC AR4 (Solomon et al. 2007), the contribution of non-frozen terrestrial waters including GWD to sea level variation is not considered due to its perceived uncertainty (Wada 2016).

GRACE (Gravity Recovery and Climate Experiment) satellite observations (Tapley et al. 2004; Chen et al. 2016) open a path to monitor total water storage changes including groundwater in data scarce regions (hereafter referred to as *“satellite-based method”*) (Strassberg et al. 2007; Jacob et al. 2012; Shamsudduha et al. 2012; Voss et al. 2013). Rodell et al. (2009) and Tiwari et al. (2009) reported substantial GWD over Northwest India (17.7 ± 4.5 km^3^ year^−1^; 0.05 ± 0.013 mm year^−1^) and adjacent regions (54 ± 9 km^3^ year^−1^; 0.15 ± 0.025 mm year^−1^) (see also Table 1). Coarse spatial resolution and noise contamination inherent in GRACE data hinder global application of estimating GWD (*satellite-based method*). Van Dijk et al. (2014) used a data assimilation framework to integrate water balance estimates derived from GRACE satellite observations and several hydrological models, which improved the estimate of global GWD derived from a hydrological model from 168 to 92 km^3^ year^−1^ (averaged over the 2003–2012 period).

**Table 1:** Global and regional estimates of groundwater depletion (GWD) contribution to sea level rise (SLR)

		GWD (km^3^ year^−1^)	SLR (mm year^−1^)	Year	Notes	Sources
Global	Sahagian et al. (1994)	86.7	0.24	Contemporary	Limited regions (e.g., USA, India, China)	Literature Country statistics
	Postel (1999)	200	0.56	Contemporary	Global	Literature Country statistics
	Gornitz (2001)	36–108	0.1–0.3	Contemporary	Limited regions (e.g., USA, India, China)	Literature Country statistics
	Foster and Loucks (2006)	26.8	0.075	Contemporary	Limited regions (e.g., Middle East, Northern Africa)	Literature Country statistics
	Wada et al. (2010)	126 (±32) 283 (±40)	0.35 (±0.09) 0.79 (±0.11)	1960 2000	Depletion equals abstraction in excess of recharge	IGRAC-GGIS PCR-GLOBWB (0.5°)
	Konikow (2011)	145 (±39)	0.4 (±0.11)	2000–2008	Extrapolated for other than USA, north India, North China Plain, Saudi Arabia, Nubian and Sahara	In situ groundwater level measurements, GRACE satellite observation, calibrated groundwater model, extrapolation (15.4 %; depletion to abstraction ratio of USA)
	Wada et al. (2012)	163 (±28) 204 (±30)	0.45 (±0.07) 0.57 (±0.08)	1990–2000 2000	Corrected against reported regional depletion estimates	IGRAC-GGIS PCR-GLOBWB (0.5°)
	Pokhrel et al. (2012)	455 (±42)	1.27 (±0.12)	2000	Depletion equals water demand in excess of water availability	MATSIRO (1.0°)
	Döll et al. (2014)	113	0.31	2000–2009	Application of deficit irrigation	WaterGAP (0.5^o^), In situ groundwater level measurements, GRACE satellite observation
	Van Dijk et al. (2014)	92	0.26	2003–2012	Without data assimilation, original depletion equals 168 km^3^ year^−1^	Data assimilation with GRACE satellite observation
	Famiglietti (2014)	77.4	0.22	2003–2013	Time periods vary among studies considered Limited regions	Various studies using GRACE-derived total terrestrial water storage changes
	Pokhrel et al. (2015)	330	0.92	2000	570 km^3^ year^−1^ for the global groundwater abstraction for 2000	MATSIRO (1.0°)
	Wada et al. (2016)	7.2 (±1.4) 97 (±14)	0.02 (±0.004) 0.27 (±0.04)	1900 2000	Coupled atmosphere-land–ocean model simulation	NCAR CESM-CAM4-CLM4 (1.0°)
Regional Northwest Sahara	Richey et al. (2015)	2.7	0.008	2003–2012	Algeria, Libya, Tunisia	GRACE-derived total terrestrial water storage changes
Middle East and North Africa (MENA)	Foster and Loucks (2006)	26.8	0.0075	Contemporary		Literature Country statistics
	Voss et al. (2013)	13.0 (±1.6)	0.036 (±0.005)	2003–2009	Cumulative 91.3 (± 10.9) km^3^ for 2003–2009	GRACE-derived total terrestrial water storage changes
Arabian	Richey et al. (2015)	15.5	0.04	2003–2013	Iraq, Jordan, Oman, Qatar, Saudi Arabia, UAE, Yemen	GRACE-derived total terrestrial water storage changes
Guarani	Richey et al. (2015)	1.0	0.003	2003–2013	Argentina, Brazil, Paraguay, Uruguay	GRACE-derived total terrestrial water storage changes
North China Plain (NCP)	Cao et al. (2013)	4.0 2.5 4.0 2.0 7.0 4.0	0.01 0.007 0.01 0.006 0.02 0.01	1960–2008 1970s 1980s 1990–1996 1997–2001 2002–2008	Cumulatively 158 km^3^ for 1960–2008 (20% of pumpage of 807 km^3^)	MODFLOW
	Feng et al. (2013)	8.3 (±1.1)	0.02 (±0.03)	2003–2010	2.5 km^3^ year^−1^ for shallow aquifers reported by Groundwater Bulletin of China Northern Plains	GRACE-derived total terrestrial water storage changes
	Huang et al. (2015)	2.5 (±0.4)-PP 1.5 (±0.2)-ECP	0.007 (±0.001) 0.004 (±0.0005)	2003–2012	Piedmont Plain (PP) East Central Plain (ECP)	GRACE-derived total terrestrial water storage changes
Indus	Cheema et al. (2014)	31	0.09	2007	68 km^3^ of total groundwater abstraction	Remote sensing combined with a hydrological model and spatial information on canal water supplies
Northern India	Rodell et al. (2009)	17.7 (±4.5)	0.05 (±0.01)	2002–2008	Cumulative 109 km^3^ for 2002–2008	GRACE-derived total terrestrial water storage changes
Northern India and surrounding regions	Tiwari et al. (2009)	54 (±9)	0.15 (±0.03)	2002–2008		GRACE-derived total terrestrial water storage changes
	Jacob et al. (2012)	35	0.1	2003–2010		GRACE-derived total terrestrial water storage changes
Bangladesh	Shamsudduha et al. (2012)	0.44(±1.24)–2.04(± 0.79)- wet seasons 0.52(± 0.5)–2.83(± 0.42)-annual	0.001(± 0.004)–0.006(± 0.002) 0.002(± 0.001)–0.008(± 0.001)	2003–2007	Depletion of 0.52 (± 0.30)–0.85 (± 0.17) km^3^ year^−1^ from borehole hydrographs	GRACE-derived total terrestrial water storage changes
California’s Central Valley	Famiglietti et al. (2011)	3.1 (± 0.6)	0.009 (± 0.002)	2003–2010	Cumulative 20.3 km^3^ for 2003–2010	GRACE-derived total terrestrial water storage changes
	Scanlon et al. (2012a)	2.0 6–8	0.006 0.017–0.022	1962–2003 2006–2010	Cumulative 24.6 km^3^ for 1976–1977, 49.3 km^3^ for 1987–1992, 140 km^3^ since the 1860s, and 80 km^3^ since the 1960s	MODFLOW
	Scanlon et al. (2012b)	8.9 (± 0.9)	0.025 (± 0.0025)	2006–2010	Cumulative 31.0 (± 3.0) km^3^ for 2006–2010	GRACE-derived total terrestrial water storage changes
High Plains Aquifer	Scanlon et al. (2012a)	5.7 7.0 12.5	0.016 0.02 0.035	1950–2007 1987–2007 2003–2013	Cumulative 330 km^3^ after pre-development in the 1950s	MODFLOW
Canning Basin	Richey et al. (2015)	3.6	0.01	2003–2013	Australia	GRACE-derived total terrestrial water storage changes

Earlier estimates of GWD contribution to SLR ranges from 0.075 to 0.30 mm year^−1^ (Sahagian et al. 1994; Gornitz 1995, 2001; Foster and Loucks 2006; see also Table 1). These studies evaluate direct groundwater storage changes but only cover a limited number of regions of the world. Using a global-scale hydrological model (hereafter referred to as “*flux*-*based method”*), Wada et al. (2010) estimated the current rate of global GWD to be 283 (±40) km^3^ year^−1^ (0.8 ± 0.1 mm year^−1^), responsible for 25 (±3) % of recently observed SLR, which is increased substantially from 126 (±32) km^3^ year^−1^ (0.35 ± 0.09 mm year^−1^) in 1960. The *flux*-*based method* provides an upper bound of GWD as it does not account for increased capture due to decreased groundwater discharge and enhanced recharge from surface waters (Bredehoeft 2002). A subsequent study of Wada et al. (2012b) applied a correction factor to remediate the overestimation and to constrain the original GWD estimate using regionally reported numbers and estimated that the average global GWD rate amounts to 163 (±28) km^3^ year^−1^ during 1990–2000, equivalent to a SLR of 0.46 (±0.08) mm year^−1^ Wada et al. (2012b) estimated that the contribution of GWD to global sea level increased from 0.035 (±0.009) to 0.57 (±0.09) mm year^−1^ during the 20th century and projected that this would increase to 0.82 (±0.13) mm year^−1^ by 2050. Döll et al. (2014) used hydrological modeling, well observations, and GRACE satellite gravity anomalies to estimate a 2000–2009 global GWD of 113 km^3^ year^−1^ (0.314 mm year^−1^) (see Table 1). Pokhrel et al. (2015) used an integrated hydrological model (*flux*-*based method*), which explicitly simulates groundwater dynamics and pumping within a global land surface model, to estimate a global GWD of 330 km^3^ year^−1^ (0.92 mm year^−1^) for year 2000. The later study overestimated GWD by calculating the difference of water demand and water availability without using groundwater pumping and recharge data. A volume-based study by Konikow (2011) estimated global GWD to be 145 (±39) km^3^ year^−1^ (0.41 ±0.1 mm year^−1^) based on measurements of changes in groundwater storage from in situ observations, calibrated groundwater modeling, GRACE satellite data, and some extrapolations using the fixed ratio of depletion to abstraction observed in the USA (15.4%).

**Fig. 1:**
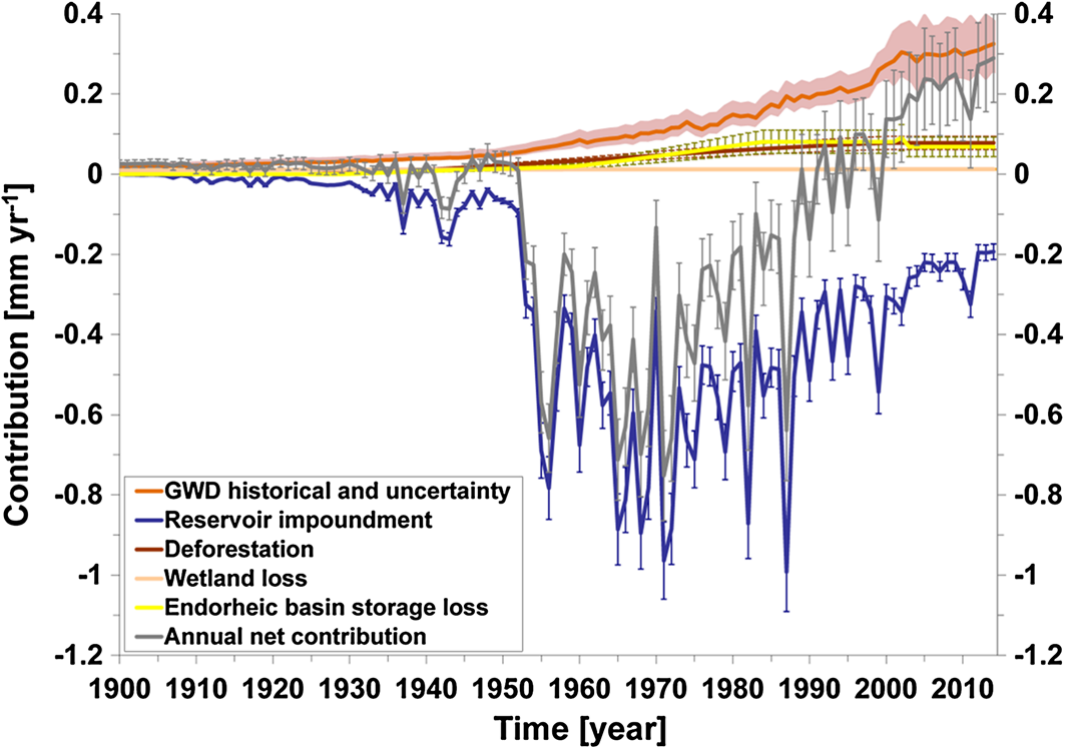
Time series of the estimated annual contribution of terrestrial water storage change to global sea level over the period 1900–2014 (rates in mm year^−1^) (modified from Wada et al. 2016)

One critical assumption of most existing global estimates of GWD impacts on sea level change is that nearly 100% of the GWD ends up to the ocean, assuming all other stores (atmospheric moisture and surface waters) to remain constant. However, groundwater pumping can also perturb regional climate due to land–atmosphere interactions (Lo and Famiglietti 2013). Over the Ogallala Aquifer in the Great Plains (USA) groundwater-fed irrigation enhances regional precipitation by 15–30% during July from the easternmost part of the aquifer to as far downwind as Indiana (DeAngelis et al. 2010) and a downwind precipitation by 20–30% over the Midwest (Kustu et al. 2010, 2011). The latest study by Wada et al. (2016) used a coupled climate-hydrological model simulation to track the fate of water pumped from underground, and found that the fraction of GWD that ends up in the ocean is 80%, over which roughly two-thirds result from an increase in runoff to the ocean, while the remainder results from the enhanced net flux of precipitation minus evaporation over the ocean. They estimated that the contribution of GWD to global SLR amounts to 0.02 (±0.004) mm year^−1^ in 1900 and increased to 0.27 (±0.04) mm year^−1^ in 2000 (Fig. 1). This indicates that most studies likely overestimated the cumulative contribution of GWD to global SLR during the 20th century and early 21st century by 5–10 mm.

## Water Impoundment Behind Dams

To acquire the history and the amount of anthropogenic water impoundment is a non-trivial task. An open depository of worldwide reservoir information is the ICOLD (International Commission On Large Dams; http://www.icold-cigb.org/) registry, which is “complete” to the extent of contributions from willing countries and water authorities. Based on the ICOLD registry and augmented with various other partial sources, Chao et al. (2008) compiled a dataset of 29,484 named artificial dams down to numerous ones with nominal water capacity less than 0.01 km^3^ the tally of which inevitably becomes grossly incomplete. They found a power law relationship between reservoir number count and reservoir capacity, to the power of −0.52, which assures that the smaller reservoirs contain negligible amount of water in global sea level contribution. Using this relationship, they estimated a total global impounded water volume of 8300 km^3^. This estimate was updated slightly by Wada et al. (2012b). In parallel, Lehner et al. (2011) introduced the Global Reservoir and Dam database (GRanD) that contains information for 6862 dams and their associated reservoirs with a total storage capacity of 6197 km^3^. By extrapolation, they estimated a global storage capacity of 8070 km^3^.

We make yet another updated database by combining the available sources after applying careful quality control. The result is a list of 48,064 reservoirs that have a combined capacity of 7968 km^3^. This new estimate included all reservoirs with a capacity larger than 1 km^3^, plus all reservoirs down to smaller than 0.01 km^3^ adopted from the ICOLD registry. The database also includes geographic locations for most of 144 reservoirs exceeding 10 km^3^ that have a combined capacity of 4331 km^3^ (see Supplementary Information). Our estimate is somewhat smaller than the estimate in Chao et al. (2008) because some reservoirs were found to be duplicated.

**Fig. 2:**
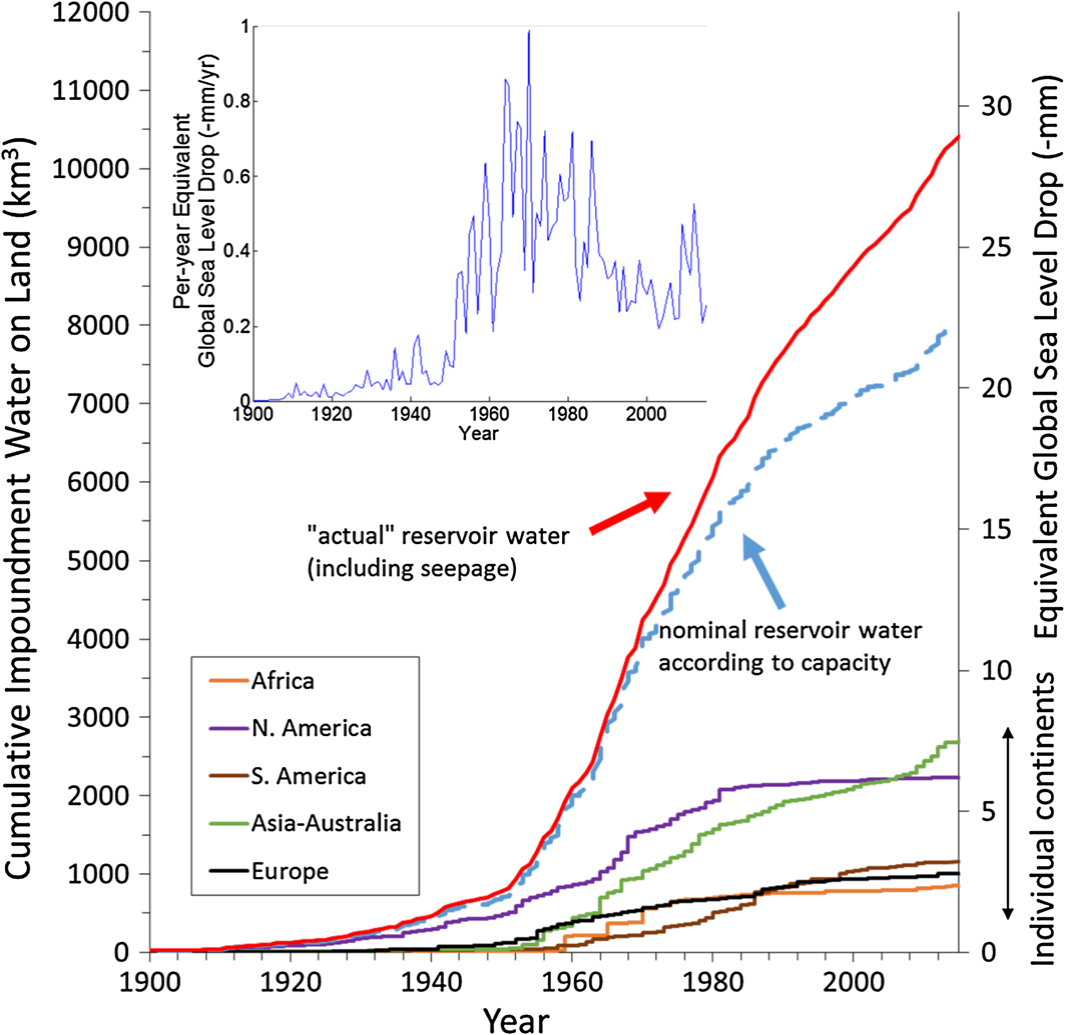
Cumulative amount of water impoundment in artificial reservoirs as a function of time during the last century, with the equivalent sea level drop on the right-hand scale. The blue dashed curve is the nominal capacity, and the red curve is the “actual” impoundment (*see text*). Also shown is the continental breakdown. The *inset* is the per-year water impoundment

Figure 2 gives the time history of the growth of the total capacity according to the nominal years of completion for each reservoir, along with a continental breakdown. Also shown is the per-year capacity, which clearly reflects the history of the human activity in dam building. From the design capacity of the reservoir to the actual amount of water impoundment, we make assumptions following Chao et al. (2008) that: (1) No reservoir is kept at full capacity at all times. The actual percentage of water storage varies from one climatic regime to the next, most typically from season to season as well as interannually. Here we assume that on average 85% of the design capacity is used. (2) There is additional water impounded by the reservoirs that seeps underground to raise the local groundwater level. This additional water is estimated to be 5% of the capacity for the first year and continues to increase slowly according to the square-root of time since completion until reaching twice of the design capacity. Certain large reservoirs are excluded from this additional water seepage because they are not “new” reservoirs but rather dam-raised lake levels of existing lakes, including the Owen Falls/Lake Victoria (the largest in the list) and several large water projects (Table 2). Lake Victoria should no more be considered as an artificial added capacity, since this lake stored 2.5 m in the early sixties (around 170 km^3^), but this was progressively lost in the following years. The actual water retention varies greatly depending on regional geology; our modeling may represent upper limits in some cases. (3) Reservoirs suffer from silt accumulation with time. That presents serious problems for water management, but has little consequence, at least to the first order, on anthropogenic contributions to sea level change as the impounded water is replaced by an equal volume of silt that would have otherwise been deposited in the ocean. In known cases, the disappearance of natural floods due to reservoirs may even encourage more silt deposit along river banks (Chao et al. 2008).

**Table 2:** Large reservoirs that are excluded from this additional water seepage due to that they are not “new” reservoirs but rather dam-raised lake levels of existing lakes, including the Owen Falls/Lake Victoria (the largest in the list) and several large water projects

Country	Reservoir name
Canada	Manicouagan
	Jenpeg
	Smallwood Reservoir
	Missi Falls Control
	Ear Falls Dam
	Whitesand Rapids
	Pipmuacan
	Keenleyside
China	Sanhezha
Finland	TainionkoskI
Russia	Irkutsk
	Verkhne-Tulomskaya
	Onda
	Kumskaya
	Verkhne-Svirskaya
USA	Structure 308

Using the new reservoir database and applying the stated assumption (presented in Fig. 2), we estimate that humans have impounded 10,416 km^3^ of water behind dams, accounting for 28.9 mm drop in global mean sea level. During the second half of the 20th century, when the dam-building activity was at its highest, the average rate of sea level change was −0.51 mm/year and is significant in comparison with other natural and anthropogenic sources of sea level change. It has been noted (Chao et al. 2008) that water impoundment can largely account for the slowing in the rate of SLR observed during the second half of the 20th century.

## Deforestation, Wetland Degradation, and Lake Storage Changes

### Deforestation/Afforestation

At present, global deforestation is a net consequence of tropical forest loss and temperate-boreal forest gain (FAO 2015; Keenan et al. 2015; MacDicken 2015; Sloan and Sayer 2015). Net deforestation releases carbon and water stored in both biotic tissues and soil, which leads to positive SLR through three primary processes. First, forest clearing eliminates evapotranspiration and thus increases total surface runoff in the hydrological budget (Meherhomji 1991, Milly et al. 2010). Gornitz et al. (1997) estimated an equivalent of 0.13 mm year^−1^ SLR attributed to deforestation-induced runoff increase in the 1980s, yet this estimate may overshoot the contribution of net global deforestation as it excludes synchronous temperate forest regrowth and restoration. The global deforestation rate has slowed in the past two decades (FAO 2015; Sloan and Sayer 2015). Based on the net deforestation rate of 3.3 million ha year^−1^ between 2010 and 2015 (FAO 2015), we here apply the same method proposed by Gornitz et al. (1997) and update a SLR equivalent of no more than 0.03 mm year^−1^ attributed to the present deforestation-induced runoff increase.

Oxidation of deforested biomass (i.e., carbohydrates in dry matter) produces carbon dioxide (CO_2_) and water, along which additional water stored within plants (except dry matter) is also released. Based on a bulk forest dry-to-wet mass ratio of 0.25 (Rohrig 1991), Gornitz et al. (1997) calculated this combined contribution from net global deforestation in the 1980s to be ~0.02 mm year^−1^ SLR. Sahagian et al. (1994), Sahagian (2000) and Vörösmarty and Sahagian (2000) further took into account an equal amount of water stored in soil, fallen leaves and surrounding atmosphere and estimated 0.14 mm year^−1^ SLR resulting from tropical deforestation alone from 1940 to 1990. Given the net loss of 0.2 Gt year^−1^ in forest carbon stock from 2010 to 2015 (FAO 2015) and assuming that such carbon loss is completely emitted, we estimate that water release from recent global deforestation, combining the amounts from both oxidation and plant storage, likely dropped to 1.5 Gt year^−1^, equivalent to less than 0.005 mm year^−1^ SLR.

Widespread deforestation also triggers complex climate feedbacks, as suggested by both modeling and observation-based studies (Butt et al. 2011; Chagnon and Bras 2005; Nobre et al. 2009; Shukla et al. 1990; Spracklen et al. 2012). Reduced evapotranspiration may suppress regional precipitation and thus groundwater recharge (Huntington 2008; Spracklen et al. 2012), counteracting the contribution of increased runoff to SLR. Deforestation-induced emission of CO_2_ and water vapor, both major greenhouse gases, leads to additional global warming (Ciais et al. 2013), which accelerates glacier and ice cap melting and reinforces SLR. Nevertheless, contributions of these climate feedbacks are coupled with and attenuated by other carbon sinks. For example, about half of the elevated CO_2_ concentration from anthropogenic emissions since 1980s, including deforestation, was taken up by ocean and terrestrial biosphere (Khatiwala et al. 2009; Sabine et al. 2004). If uncertainties from the land-atmospheric coupling are excluded, we suggest that the current net global deforestation, synthesizing the direct impacts of runoff increase and water release from oxidation and plant storage, leads to an upper-bound SLR of ~0.035 mm year^−1^.

### Wetland Degradation

Wetland degradation contributes to SLR primarily through (1) direct water drainage or removal from standing inundation, soil moisture, and plant storage and (2) water release from vegetation oxidation and peat combustion. The latter process is similar to that of deforestation (Sect. 5.1), except that additional water is released from the burning of peat harvested as an important fuel source in certain regions (van der Werf et al. 2010). The scale of wetland contribution to SLR remains poorly constrained, largely because of our incomplete knowledge of global wetland changes. There is still no universal consensus on the definition of wetland (Mitra et al. 2005) due to the diversity of wetland types. This introduces fundamental ambiguity in quantifying wetland extents and water/carbon storage. Thus, a major advancement of global wetland inventory and monitoring is needed to reduce uncertainties in estimates of their contributions to SLR.

Preliminary estimates were inferred by several studies from regional wetland records in the past two centuries. Sahagian et al. (1994) calculated that, in the USA alone, wetland drainage since 1780 had led to an average SLR of 0.006 mm year^−1^. This estimation was based on a documented wetland loss of 0.22 million ha (mha) year^−1^ in the USA (Mitsch and Gosselink 1993) and an assumed ~1 m water column depth. Milly et al. (2010) assumed a global wetland area of 856 mha based on Mitsch and Gosselink (1993) and a 50% area loss of the original wetland. By spreading that drainage over the same 220 years, they obtained a global contribution of ~0.06 mm year^−1^ SLR, 10 times of that of the USA. Davidson (2014) recently argued that the presumed global wetland loss of 50% could be substantially underestimated. By synthesizing 189 reports of wetland area changes, this analysis shows that 71% of the original natural wetlands that fall under the Ramsar Convention for wetland types (Matthews 1993) had been lost by early 21st century, and 67% was lost during past two centuries, implying that the estimate of Milly et al. (2010) is likely conservative. Here we consider a recent wetland loss rate of 0.565% year^−1^ since 1990 (Davidson 2014) and a present global wetland area of 371 mha averaged from three databases: Matthews natural wetlands (Matthews and Fung 1987), ISLSCP (Darras 1999), and DISCover (Belward et al. 1999; Loveland and Belward 1997). If we also assume a uniform 1 m depth of water in wetlands (Milly et al. 2010), the contribution of recent global wetland drainage to SLR would be 0.067 mm year^−1^.

The assessments above, however, exclude the impacts of biomass oxidation and peat combustion. According to Armentano and Menges (1986) and Gornitz et al. (1997), the net global carbon emission from 1795 to 1980, integrating carbon storage reduction by peat combustion and in temperate and tropical wetlands sums to 22–101 million t C year^−1^. Water release associated with such carbon emission was converted by Gornitz et al. (1997) similarly to that of deforestation (Sect. 5.1), yielding a SLR of 0.001–0.002 mm year^−1^. Inferred from multiple sources, Mitra et al. (2005) inferred from multiple sources that global mean carbon densities are 210–700 t C ha^−1^ in wetland soils (including peatland) and ~50 t C ha^−1^ in vegetation biomass. If we apply the same wetland area and loss rate as used for assessing wetland water drainage, the annual reduction of wetland carbon stock since 1990, if completely emitted, releases water equivalent to 0.003–0.007 mm year^−1^ SLR. Integrating the impacts of wetland drainage, oxidation and peat combustion, we here suggest that the recent global wetland degradation results in an upper-bound SLR of ~0.075 mm year^−1^.

### Lake Storage Changes

Lakes store the greatest mass of liquid water on the terrestrial surface (Oki and Kanae 2006). Variation in lake water storage shares an intrinsic bond with that of the entire terrestrial water storage that links directly to SLR (Reager et al. 2016). Compared to some other storage forms such as glaciers and groundwater, lakes have more active interactions with surface and land–atmosphere fluxes, thus typically more dynamic in budget (Sheng et al. 2016; Song et al. 2014; Wang et al. 2012). In many regions of the world, lakes are monitored as “sentinels” of both climate change and anthropogenic impacts (Adrian et al. 2009; Smith et al. 2005; Song et al. 2016; Wang et al. 2014). However, also because of this “dynamic” nature, along with lakes’ extensive distribution in various environments, changes in lake water storages on a global scale are poorly known, and their overall contribution to SLR remains unclear.

Existing studies of lake storage contributions in recent decadal and centennial periods focus on big endorheic (landlocked) lakes, whose growth and declines likely signal the opposite loss and gain in the exorheic (ocean connected) storage. In the past century, perhaps the greatest contributor in global lake storage was the largest endorheic lake, the Caspian Sea (Milly et al. 2010), where water level exhibits substantial oscillations attributed to meteorological, geological, and anthropogenic factors (Ozyavas et al. 2010). A centennial record of the Caspian Sea level, measured by both gauging stations and recent satellite altimetry (Cretaux et al. 2011; Golytsyn and Panin 1989; Klige and Myagkov 1992; Schwatke et al. 2015), reveals an enduring drop of ~3 m during 1900–1977 inducing 0.05 mm year^−1^ SLR and a ~2-m increase in the subsequent two decades or –0.12 mm year^−1^ SLR (Milly et al. 2010), followed by another drop of 0.722 (±0.026) m from 1995 to the near present (year 2014) or, as we calculate here, 0.047 (±0.002) mm year^−1^ SLR. If we assume that the lake level variation kept pace with groundwater changes which can be approximated using the method proposed by Sahagian et al. (1994), the overall contribution of the Caspian Sea, including both surface and groundwater storage variations, has been about 0.03 mm year^−1^ SLR since 1900, 0.075 (±0.002) mm year^−1^ since 1995, or 0.109 (±0.004) mm year^−1^ since 2002.

In contrast to Caspian Sea’s level fluctuations, the Aral Sea has been falling constantly over the past half a century. Between 1960 and 1990, the water storage in the Aral Sea Basin declined at a striking rate of 64 km^3^ year^−1^, equivalent to 0.18 mm year^−1^ SLR (Sahagian 2000; Sahagian et al. 1994; Vörösmarty and Sahagian 2000). The main culprit was attributed to be upstream water diversion for irrigation (Perera 1993), which was modeled by Pokhrel et al. (2012) to be ~500 km^3^ during 1951–2000, equivalent to 0.03 mm year^−1^ SLR. This modeled irrigation-induced contribution is substantially smaller than the estimate of Sahagian et al. (1994) which accounts for basin-scale water storage changes inferred from historical documentation (Micklin 1992). Such a discrepancy likely implies the importance of other factors such as enhanced land-to-atmospheric flux and climate variations. Dramatic decline in the Aral Sea continued in the recent decade, with an annual rate of 6.043 (±0.082) km^3^ year^−1^ measured from 2002 to 2014 (Schwatke et al. 2015). If we assume that groundwater drainage has kept pace with lake level reduction (Sahagian et al. 1994), the Aral Sea has contributed to the recent SLR by 0.0358 (±0.0003) mm year^−1^.

In the recent couple of decades, advancement of altimetric and gravimetric satellites has enabled more extensive and frequent monitoring of lake water storage all over the world. Evident level and volume decrease after 2000 was revealed in major lakes along East Africa’s Great Rift Valley, such as Lakes Victoria, Tanganyika, and Malawi (Ahmed et al. 2014; Awange et al. 2008; Becker et al. 2010; Ramillien et al. 2014; Swenson and Wahr 2009). Several large lakes in the Middle East, such as the Aral Sea and Urmia Lake, continued with striking water storage declines in early 21st century driven by climate change and human water diversion (Cretaux et al. 2011; Schwatke et al. 2015; Singh et al. 2012; Tourian et al. 2015). On the contrary, most endorheic lakes across High Asia exhibited rapid expansions due to wetting climate and increasing glacier meltwater supply (Song et al. 2013; Zhang et al. 2013), which resulted in a small negative contribution to SLR (Jacob et al. 2012). The endorheic Lake Chad in Africa, despite substantial shrinkage and desiccation in the 20th century, has experienced a steady budget recovery of 0.05–0.06 m year^−1^ since the 1990s (Schwatke et al. 2015), counteracting the recent SLR but by a trivial amount (~0.0002 mm year^−1^). As Milly et al. (2010) suggested, lake water storage is dominated by regional interannual variation, and thus trends cannot be extrapolated beyond the study periods or areas. A holistic and continuous understanding of its overall contribution to global SLR requires real-time observations of detailed variations in surface water volume, which fortunately are being approached by some of the near-future satellites such as NASA’s Surface Water and Ocean Topography (SWOT) mission.

## Climate-Driven Water Storage Change

Interannual to decadal changes in the sea level budget are important in understanding long-term changes in rates of SLR. The rate of SLR is strongly influenced by the transfer of water between ocean and land, the rate of which changes in response to internal climate variability such as the Pacific Decadal Oscillation (PDO) and the El Niño Southern Oscillation (ENSO), mountain glacier changes, human management practices, and to climatic change in atmospheric transport and delivery of moisture to the continents. Understanding this short-term variability is important so that the longer-term observational sea level record can be correctly interpreted and that the detection and attribution of underlying trends can be improved. A comprehensive understanding of global SLC (sea level change) depends upon a strong understanding of land water storage and its variability.

The term “climate-driven land water storage” can be used to describe variability in global hydrology and water storage in several states, including global snow, surface water, soil moisture, and groundwater storage. These sources comprise what is acknowledged to be one of the most important components of decadal sea level budgets, but also one of the most difficult to observe and characterize globally (Church et al., 2013). The annual cycle of land water storage represents an amplitude of 17 ± 4 mm sea level equivalent (SLE) of water mass, moved through the seasonal distribution of water from ocean to land (e.g., Wouters et al. 2011). Because of this large-amplitude oscillation, natural changes in the interannual to decadal cycling of water can have a large effect on the apparent rate of SLC over decadal and shorter time periods (Milly et al. 2003).

**Fig. 3:**
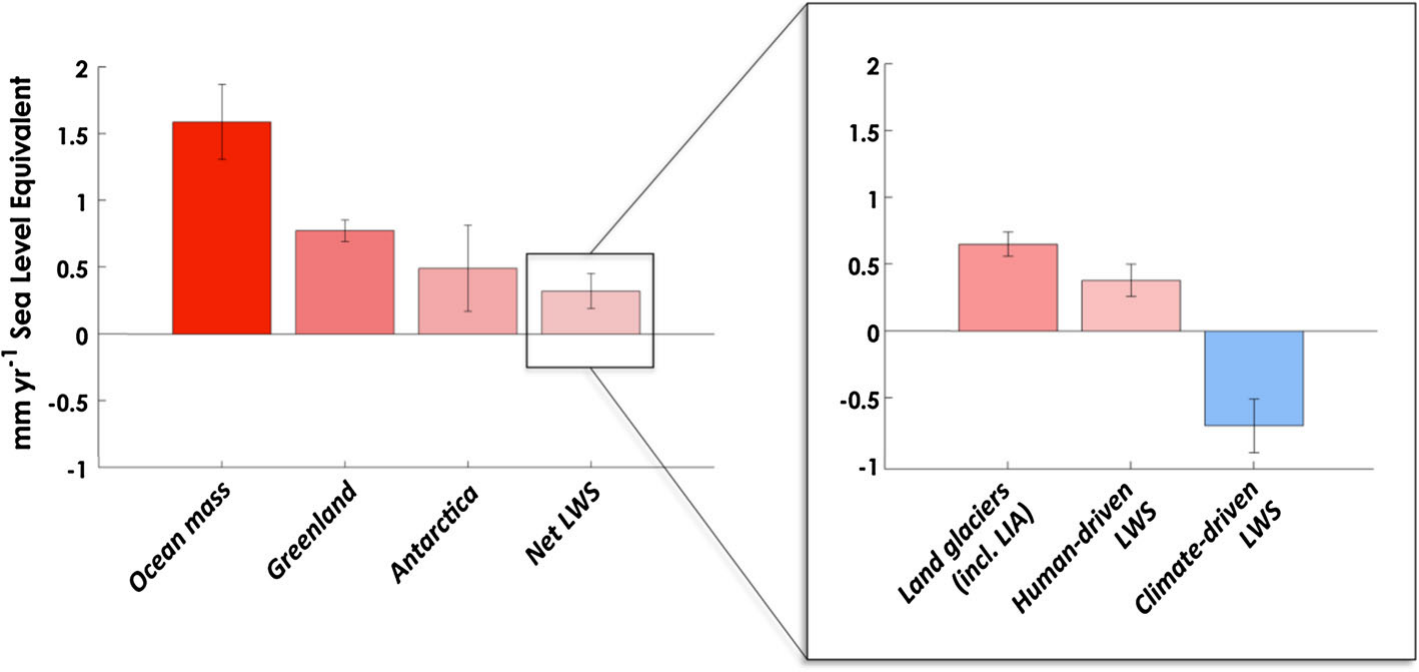
Global mass budget estimate from Reager et al. (2016). This includes a disaggregation of the land water storage estimate into land glaciers, human-driven, and climate-driven water storage

From the years 2003–2011, the altimetry reported rate of SLR was ~2.4 mm year^−1^ (Cazenave et al. 2014). However, increased mass loss from glaciers (Gardner et al. 2013) and ice sheets (Shepherd et al. 2012) during this time period made this lower rate difficult to reconcile with component-based SLC budgets. Recent research using observations from NASA’s GRACE satellite mission to detect changes in global land and ocean mass identified climate-driven changes in land water storage as the major contributor to the apparent decrease in the rate of SLR (i.e., large net transfer from ocean to land, Reager et al. 2016). Reager et al. (2016) estimated a total continental land mass change (including glaciers) over their 2002–2014 study period of 0.32 ± 0.13 mm year^−1^ of SLR (i.e., ocean gaining). This result compares well with trends in land and ocean mass change in studies by Riva et al. (2010), Llovel et al. (2010), (2014), and Jensen et al. (2013) when differences in time periods are accounted for. To isolate a hydrology-only “land water storage” signal, Reager et al. (2016) then removed global land glacier mass loss trends. Estimating a glacier rate of 0.65 ± 0.09 mm year^−1^ of SLR (ocean gaining), glacier-free land gained or stored water at a rate of 120 ± 60 Gt year^−1^ equivalent to –0.33 ± 0.16 mm year^−1^ of SLR (i.e., land gaining). Increases in precipitation over land caused positive storage trends in some regions: e.g., large flooding periods in the upper Missouri River basin (Reager et al. 2014); recovery from drought in the Amazon (Chen et al. 2010), the Zambezi and Niger basins in Africa (e.g., Ramillien et al. 2014), and weaker gains in Northern Australia associated with La Niña (Fasullo et al. 2013). Using the GRACE observations to represent the net land water storage mass gain (equivalent to –0.33 ± 0.16 mm year^−1^ of SLR) over land (i.e., the sum of both the human- and climate-driven components), subtracting the IPCC estimate for the human-driven component of 0.38 ± 0.12 mm year^−1^ of SLR (Church et al. 2013) provides an estimate of the climate-driven land water storage change. Applying this method, an estimated –0.71 ± 0.20 mm year^−1^ SLE of land water storage uptake is required to close the observed land water storage balance. This number agrees well with the hypothesis and numbers presented by Cazenave et al. (2014) who provided much lower SLR estimate over a recent decade (Table 3) and supports the concept that ENSO-driven modulations of the global water cycle are of first-order importance in decadal-scale sea level budgets, roughly comparable to the magnitudes of ice mass losses from glaciers and ice sheets (Fig. 3).

**Table 3:** Global land water storage budget (2002–2014) in mm year^−1^

		2002–2014 (mm year^−1^)
Observed SLR	Church et al. (2013) (1993–2010)	3.2 (±0.4)
	Cazenave et al. (2014) (2003–2011)	2.4
Estimated Land water storage		
Groundwater	Wada et al. (2016)	0.30 (±0.1)
Reservoir impoundment	This study	−0.24 (±0.02)
Deforestation (after 2010)	This study	0.035
Wetland loss (after 1990)	This study	0.075
Endorheic basin storage loss		
Caspian	This study	0.109 (±0.004)
Aral Sea	This study	0.036(±0.0003)
Climate-driven land water storage	Reager et al. (2016)	−0.71 (±0.2)
Net land water storage	This study	−0.40 (±0.2)
	IPCC AR5 (1993–2010)	0.38 (±0.12)

Reager et al. (2016) use a direct observation of the global land mass change, including detailed measurements of glacier change. In their approach, they provide a thorough and rigorous quantification of the uncertainty for all mass change terms over land, including not only the measurement and leakage errors, but also uncertainties in the GRACE post-processing corrections for geocenter, mean pole, and glacial isostatic adjustment (GIA), as well as the uncertainties in glacier change. Because of this, the confidence intervals presented in Reager et al. (2016) should be considered the most comprehensive assessment of uncertainties present to date. This study marks a new era in which changes in land water storage and the potential impact of climate-driven changes in hydrology can be measured from land water storage space and applied to improve the interpretation of the sea level record.

Sea level variability due to climate-driven hydrology will change with the length of the record and does not represent a long-term offset to global SLR (Dieng et al. 2015). However, over the last decade, climate-driven land water storage uptake is of opposite sign and of comparable magnitude to ice losses from glaciers and ice sheets and nearly twice as large as mass losses from direct human-driven changes in land water storage. Climate-driven changes in land water storage are now observable on a global scale and that these changes are large and necessary for closure of decadal-scale sea level budgets.

## Updated Land Water Contribution and Discussion

Based on separate contribution from different land water storage components, we estimate the net land water storage contribution to SLR is −0.40 (±0.2) during the period of 2002–2014 (Table 3). When considering terrestrial water contribution to SLR including groundwater, reservoir impoundment, water release due to deforestation, marsh drainage or wetland loss, and storage loss from endorheic lakes, it is important to note that reservoir impoundment due to dam building is of the opposite sign in its sea level contribution, suggesting that the volume of water accumulated in reservoirs up to 2015 amounts to ~30 mm sea level equivalent. Lettenmaier and Milly (2009) indicated that the volume of silt accumulated in reservoirs should be removed, which is equal to ~4 mm sea level equivalent. Indeed, silting-up of existing reservoirs may already be, or in coming decades may become, a larger effect on impoundment than the construction of new reservoir capacity (Wisser et al. 2013). It is also important to note that climate-driven land water storage change has a large negative contribution to SLR over the period 2002–2014. Together with reservoir impoundment, the overall negative contribution amounts to nearly 1 mm year^−1^ over the period 2002–2014, which cancels out all the positive contribution from groundwater, and the remaining land water storage change. However, climate-driven land water storage likely has large decadal variability over past and future periods, which are not yet accounted for in estimated sea level variations.

During the period 1900–1950, the net contribution of terrestrial water sources to global SLR is small with the average rate of +0.014 (±0.008) mm year^−1^; however, as a result of increased dam building during 1950–1990 the net contribution became consistently negative with the average rate of −0.34 (±0.025) mm year^−1^. Since the 1990s dam building has been tapering off and GWD has been steadily increasing, the net contribution became positive with the average rate of +0.12 (±0.08) mm year^−1^ over the period 1993–2010 (Wada et al. 2016). During the recent decade, GWD is the most important positive terrestrial water contribution (Wada et al. 2012). The increase is driven by growing water demand for population and agricultural production over intense irrigated regions including India, Pakistan, China, Iran, USA, Mexico, and Saudi Arabia.

For the contribution from deforestation, wetland loss, and endorheic basin storage loss, we estimate the overall contribution to be 0.22 mm year^−1^ over the period 2002–2014, which still gives a significant positive contribution (40% of the overall positive contribution). However, large uncertainty still remains and the contribution likely varies substantially due to human and climate influences over time.

Using the latest available estimates of land water contributions, we estimate the net land water contribution during the period 2002–2014 to be largely negative (−0.40 mm year^−1^). Although the time periods are not exactly the same, this suggests a large discrepancy from the estimated net land water contribution reported in the IPCC AR5 (0.38 mm year^−1^; 1993–2010).

## Electronic supplementary material

Below is the link to the electronic supplementary material.

Supplementary material 1 (XLSX 26 kb)
